# Potential role of sorafenib as neoadjuvant therapy in unresectable papillary thyroid cancer

**DOI:** 10.20945/2359-3997000000046

**Published:** 2018-05-07

**Authors:** Debora L. S. Danilovic, Gilberto Castro, Felipe S. R. Roitberg, Felipe A. B. Vanderlei, Fernanda A. Bonani, Ricardo M. C. Freitas, George B. Coura-Filho, Rosalinda Y. Camargo, Marco A. Kulcsar, Suemi Marui, Ana O. Hoff

**Affiliations:** 1 Universidade de São Paulo Universidade de São Paulo Faculdade de Medicina Instituto do Câncer do Estado de São Paulo São Paulo SP Brasil Endocrinologia, Instituto do Câncer do Estado de São Paulo, Faculdade de Medicina da Universidade de São Paulo (FMUSP), São Paulo, SP, Brasil; 2 Universidade de São Paulo Universidade de São Paulo Faculdade de Medicina Laboratório de Endocrinologia Celular e Molecular, Hospital das Clínicas São Paulo SP Brasil Laboratório de Endocrinologia Celular e Molecular (LIM25), Hospital das Clínicas, Faculdade de Medicina da Universidade de São Paulo (HCFMUSP), São Paulo, SP, Brasil; 3 Universidade de São Paulo Universidade de São Paulo Faculdade de Medicina Instituto do Câncer do Estado de São Paulo São Paulo SP Brasil Oncologia Clínica, Instituto do Câncer do Estado de São Paulo (ICESP), Faculdade de Medicina da Universidade de São Paulo (FMUSP), São Paulo, SP, Brasil; 4 Universidade de São Paulo Universidade de São Paulo Faculdade de Medicina Hospital das Clínicas São Paulo SP Brasil Cirurgia de Cabeça e Pescoço, Hospital das Clínicas, Faculdade de Medicina da Universidade de São Paulo (HCFMUSP), São Paulo, SP, Brasil; 5 Universidade de São Paulo Universidade de São Paulo Faculdade de Medicina Instituto do Câncer do Estado de São Paulo São Paulo SP Brasil Cirurgia de Cabeça e Pescoço, Instituto do Câncer do Estado de São Paulo (ICESP), Faculdade de Medicina da Universidade de São Paulo (FMUSP), São Paulo, SP, Brasil; 6 Universidade de São Paulo Universidade de São Paulo Faculdade de Medicina Instituto do Câncer do Estado de São Paulo São Paulo SP Brasil Radiologia, Instituto do Câncer do Estado de São Paulo (ICESP), Faculdade de Medicina da Universidade de São Paulo (FMUSP), São Paulo, SP, Brasil; 7 Universidade de São Paulo Universidade de São Paulo Faculdade de Medicina Instituto do Câncer do Estado de São Paulo Brasil Medicina Nuclear, Instituto do Câncer do Estado de São Paulo (ICESP), Faculdade de Medicina da Universidade de São Paulo (FMUSP), São Paulo, SP, Brasil

## Abstract

Total thyroidectomy, radioiodine (RAI) therapy, and TSH suppression are the mainstay treatment for differentiated thyroid carcinomas (DTCs). Treatments for metastatic disease include surgery, external-beam radiotherapy, RAI, and kinase inhibitors for progressive iodine-refractory disease. Unresectable locoregional disease remains a challenge, as standard therapy with RAI becomes unfeasible. We report a case of a young patient who presented with unresectable papillary thyroid carcinoma (PTC), and treatment with sorafenib allowed total thyroidectomy and RAI therapy. A 20-year-old male presented with severe respiratory distress due to an enlarging cervical mass. Imaging studies revealed an enlarged multinodular thyroid gland, extensive cervical adenopathy, severe tracheal stenosis, and pulmonary micronodules. He required an urgent surgical intervention and underwent tracheostomy and partial left neck dissection, as the disease was deemed unresectable; pathology revealed PTC. Treatment with sorafenib was initiated, resulting in significant tumor reduction allowing near total thyroidectomy and bilateral neck dissection. Postoperatively, the patient underwent radiotherapy for residual tracheal lesion, followed by RAI therapy for avid cervical and pulmonary disease. The patient's disease remains stable 4 years after diagnosis. Sorafenib has been approved for progressive RAI-refractory metastatic DTCs. In this case report, we describe a patient with locally advanced PTC in whom treatment with sorafenib provided sufficient tumor reduction to allow thyroidectomy and RAI therapy, suggesting a potential role of sorafenib as an induction therapy of unresectable DTC.

## INTRODUCTION

The treatment of differentiated thyroid carcinomas (DTCs) involves a combination of total thyroidectomy, radioactive iodine (RAI) therapy, and TSH suppression. On the other hand, treatment of metastatic DTC is more complex and includes surgery when feasible, ^131^I therapy, external beam radiotherapy, and other localized treatment procedures such as thermal ablation or stereotactic radiation. However, a few cases of metastatic carcinomas progress despite RAI treatment, and therapy with multikinase inhibitors (MKIs) becomes the alternative to control systemic disease. The MKIs can inhibit tumor growth and angiogenesis. They block molecular targets involved in the pathogenesis of thyroid cancer, such as BRAF, RET, vascular endothelial growth factor receptors (VEGFRs), platelet-derived growth factor receptors (PDGFRs), and fibroblast growth factor receptors (FGFRs). Due to clinical benefit demonstrated in phase-III clinical trials, the FDA has recently approved the use of sorafenib and lenvatinib in treatment of progressive iodine-refractory metastatic DTC (1,2).

Locally aggressive papillary thyroid carcinoma is as an unusual presentation, considering the characteristic slow growth of papillary carcinomas. Near total or total thyroidectomy is advised and necessary for effective RAI therapy; therefore, when invasive neck disease is unresectable, traditional therapy, especially RAI, becomes unfeasible.

We present a case of a patient with unresectable papillary thyroid carcinoma at presentation in whom preoperative treatment with sorafenib allowed surgical intervention and RAI therapy.

This case report was approved by the local Institutional Review Board.

## CASE REPORT

**Figure 1:**
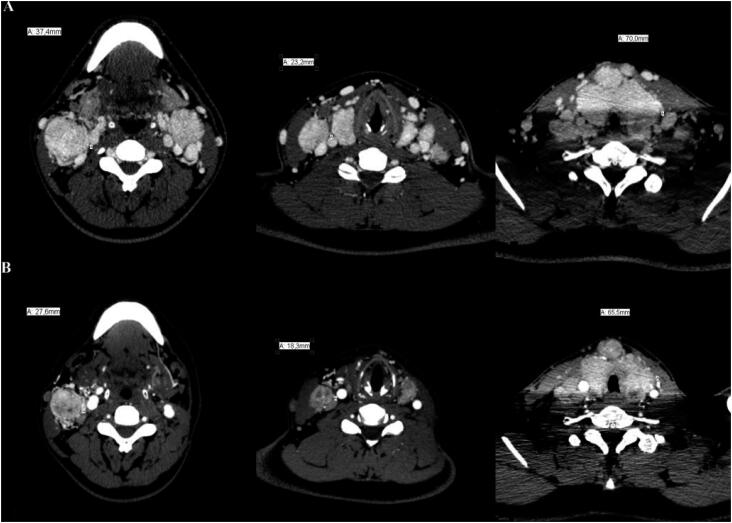
(**A**) Initial CT scans revealing thyroid enlargement with tracheal stenosis and bilateral conglomerates of cervical lymph nodes with compression of adjacent structures. (**B**) CT scans after 12 months of sorafenib therapy, demonstrating significant reduction of neck disease.

**Figure 2:**
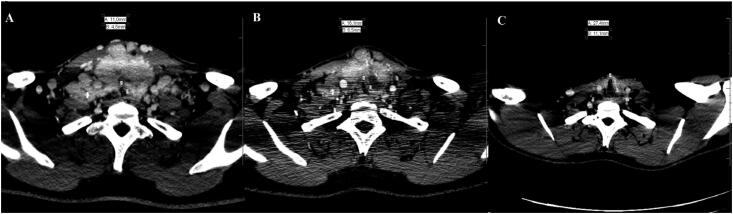
CT imaging demonstrating improvement of tracheal compression and lumen reduction. (**A**) Initial scan. (**B**) Scan after 12 months of sorafenib. (**C**) Last scan after 4 years of diagnosis.

A 20-year-old male presented at the emergency room with severe respiratory distress. He had a 4-year history of an enlarging cervical mass and dyspnea for 6 months. Imaging studies revealed a large and nodular heterogeneous thyroid with calcifications causing severe tracheal stenosis and compression of internal jugular veins, in addition to conglomerates of cervical lymph nodes and multiple, up to 1-cm, pulmonary nodules (Figure 1A, Figure 2A). The severe respiratory distress required urgent surgical intervention. During surgery, the right thyroid lobe was completely adhered to the trachea, as were metastatic right neck lymph nodes to the thyroid. Due to extensive bleeding and functional impairment of the right recurrent laryngeal nerve during intraoperative neuromonitoring, total thyroidectomy was abandoned and only left neck dissection and tracheostomy were performed; an intraoperative frozen section biopsy revealed papillary thyroid carcinoma.

External beam radiotherapy is usually the first palliative choice for unresectable thyroid carcinoma, especially in older patients, but the presence of bulky disease would not only demand high cumulative doses but also result in high toxicity and even increased risk of secondary malignancies (3). Additionally, RAI therapy would not be feasible without thyroidectomy. Therefore, after multidisciplinary discussion, the recommendation was to proceed with sorafenib treatment. The patient tolerated sorafenib at a dose of 400 mg bid with manageable side effects of fatigue, hand and foot skin reaction, and diarrhea. Reduction of the cervical mass, particularly metastatic lymph nodes, and pulmonary nodules was observed within 6 months of therapy, resulting in removal of the tracheostomy tube. Sorafenib was maintained for 13 months until significant tumoral reduction to surgery (Figure 1B, Figure 2B). Near total thyroidectomy and bilateral neck dissection were performed after discontinuation of sorafenib for 2 weeks with no complications. Pathology revealed a 7.2-cm multifocal, diffuse sclerosing papillary carcinoma with gross extrathyroidal extension, positive margins, and involvement of 13 out of 38 metastatic lymph nodes (pT4pN1bM1, stage II) (4). Genetic analysis of tumoral tissue was negative for the p.V600E *BRAF* mutation.

**Figure 3:**
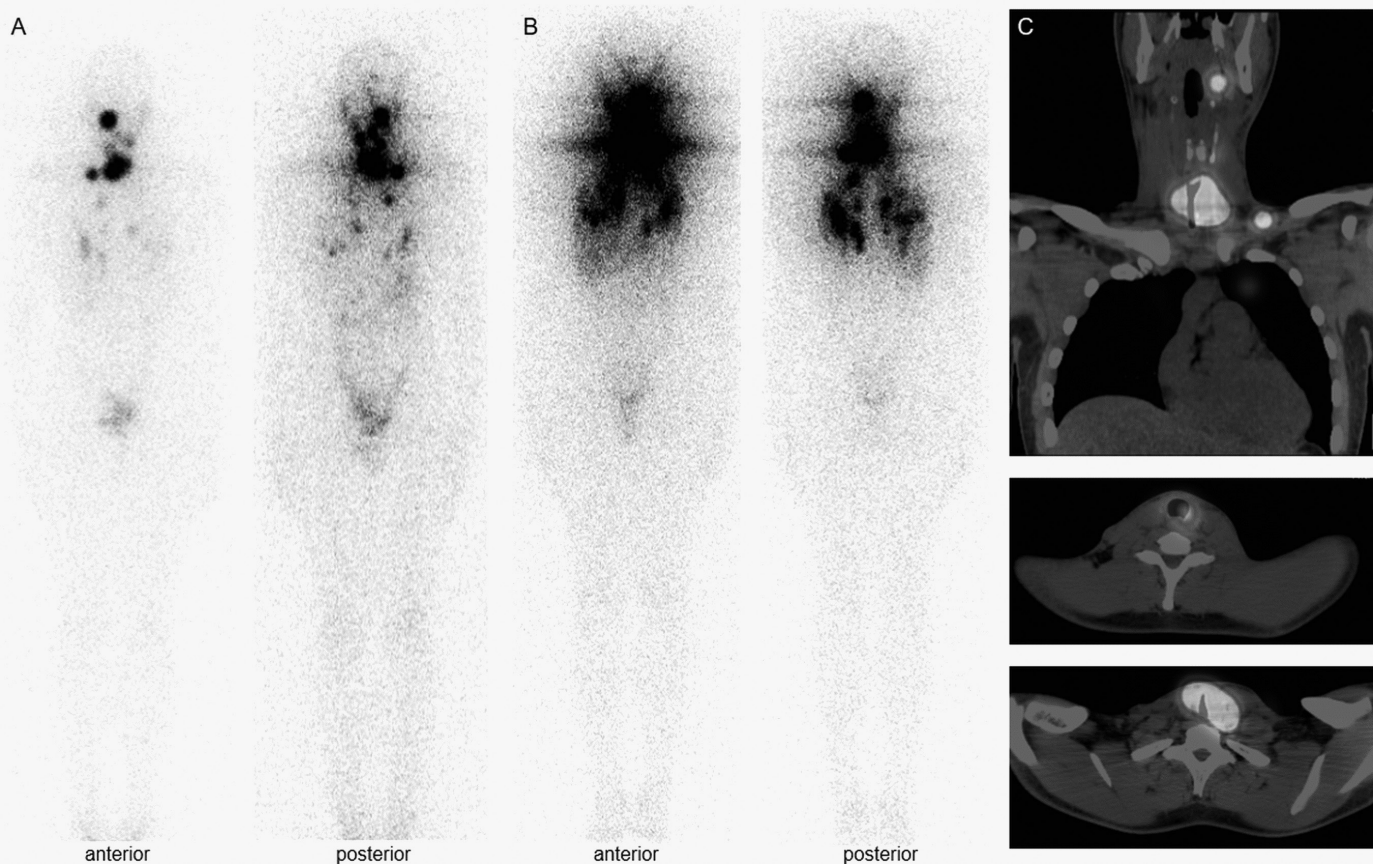
(A) Diagnostic whole-body scan after 3 months of adjuvant external beam radiotherapy. (B) Post-therapy whole-body scan revealing radioiodine-avid cervical lesions and pulmonary nodules. (C) Concomitant post-therapy single photon emission computed tomography–computed tomography (SPECT/CT) images.

The patient was placed on TSH suppression therapy; suppressed thyroglobulin was 1.5 ng/mL but with positive anti-thyroglobulin antibody (103 UI/mL). He underwent external beam radiotherapy, 70 Gy delivered in 35 fractions, for control of residual macroscopic tracheal lesion. A whole-body scan (^131^I-WBS) performed 3 months after radiotherapy revealed an uptake of 5.8% in the cervical area and iodine-avid pulmonary nodules (Figure 3A), which allowed him to be treated with 100 mCi of ^131^I. Post-dose WBS with SPECT/CT confirmed radioiodine avid lesions in the thyroid bed (with persistent but smaller tracheal compression and lumen reduction), lymph nodes of the left retropharyngeal space, right neck level III, and left neck level V and bilateral pulmonary nodules (Figures 3B and 3C). Imaging studies 16 months after RAI therapy revealed a persistent but stable lesion in the thyroid bed, neck metastatic lymph nodes up to 1.4 cm, and stable pulmonary nodules up to 1.1 cm. Suppressed thyroglobulin was undetectable (< 0.2 ng/mL), but levels of anti-thyroglobulin antibody increased (944 UI/mL). He received a second dose of RAI, 200 mCi, confirming persistent radioiodine avid lesions. Patient remains asymptomatic with stable metastatic disease after 52 months of diagnosis (Figure 2C). His suppressed thyroglobulin level is undetectable with decreasing levels of anti-thyroglobulin antibodies (455 UI/mL).

## DISCUSSION

Extensive invasive thyroid carcinoma is an uncommon initial presentation of DTC (5); however, its aggressive behavior significantly endangers prognosis. Cancer-specific mortality is 3-fold higher in the T4 stage according to the AJCC classification system (6). The 10-year disease-specific survival of thyroid carcinomas with extensive invasion of soft tissues, larynx, trachea, esophagus, or recurrent laryngeal nerve (T4a) reduces from 73% to 7.7% specifically when the tumor invades the prevertebral fascia or mediastinal vessels or encases the carotid artery (T4b) (7). Surgical removal is essential to eradicate the tumor. Residual microscopic disease can be managed with RAI therapy and TSH suppression, but macroscopic persistent disease may require other adjuvant therapy, usually external beam radiotherapy, to improve local control (8).

Indication of induction chemotherapy in inoperable DTC is not well established (9). Retrospective nonrandomized studies reported the benefit of cytotoxic agents in locally invasive follicular and papillary carcinomas prior to surgery (10,11). The studies observed tumor reduction after preoperative use of vinblastine alone, vinblastine with doxorubicin, and other regimens, including radiotherapy in 14% and 25% of the cases. Ito and cols. reported significant tumor reduction in 1 out of 2 papillary thyroid carcinomas with a squamous cell carcinoma component treated with paclitaxel before surgery (12). None of the studies reported toxicity.

This is the first description of the off-label induction use of sorafenib in locally invasive papillary thyroid carcinoma. The phase-III trial for RAI-refractory differentiated thyroid cancer (DECISION trial) included 7 patients (3.8%) with locally advanced thyroid cancer in the sorafenib arm (1), however, no specific information regarding this group of patients was provided. As inclusion criteria required RAI-refractory disease, one could assume that these patients likely had undergone thyroidectomy prior to a whole-body iodine scan and/or RAI therapy. Therefore, despite having locally advanced disease, they were likely different from our case report.

The preoperative use of MKI has been described in other cancers, alone or combined with other chemotherapies (13-15). Sunitinib has been used in an unresectable medullary thyroid cancer (16). Particularly in renal cell carcinomas, the neoadjuvant use of MKI provided 9.6% to 28.3% reduction in renal tumor diameter, and it changed unresectable to resectable tumors in around 20% of cases (13). Preferentially, an induction therapy should have high rates of tumor reduction. Despite a significant tumor shrinkage with sorafenib in our case, the objective response rate in the sorafenib phase-III trial was only 12.2%. (1). On the contrary, a phase-III trial of lenvatinib in RAI-refractory DTC led to complete response in 15% and partial response in 63% of treated patients. The median time to objective response with lenvatinib was only 2 months (17). Possibly, lenvatinib would be a more effective alternative for induction therapy of locally advanced DTC. Clinical trials are necessary to define the most suitable drug.

A major criticism of preoperative use of MKIs is wound complications. Inhibition of VEGFR impairs angiogenesis and granulation tissue formation (18). There are potential risks of severe bleeding and poor wound healing. Tracheoesophageal fistula could occur in a cervical invasive carcinoma. After a withdrawal period of 2 weeks, our patient had adequate wound healing. During previous experiences of patients on sorafenib with renal carcinomas, no major complications were observed, despite discontinuing the drug only a median of 3 days before surgery. The authors attributed the safety of preoperative use of sorafenib to its short half-life (25-48 hours) (19). There are only a few reports of wound complications with MKIs (18). Therefore, more data are necessary to establish the adequate time to withhold the drug before a surgical procedure. It is possible that the use of more selective inhibitors would avoid wound complications, such as vemurafenib for BRAF-mutated papillary thyroid carcinomas, which is under investigation (NCT01709292). In a phase-II trial of vemurafenib in patients with *BRAF*^V600E^-positive metastatic or unresectable papillary thyroid cancer refractory to radioactive iodine, researchers observed a partial response in 38.5% of treated patients (20). Although the objective response was smaller than that observed with lenvatinib (2), the drug does not present an anti-angiogenic effect that favors aerodigestive fistulas and impairs wound healing.

In this case report, we present 2 important observations. The first one is the potential use of sorafenib preoperatively for tumor reduction, permitting surgery. Surgical resection is more effective in the local control of the disease, preventing precocious airway obstruction and definite tracheostomy and reducing mortality related to invasive disease. We indeed improved the prognosis of a young patient with radioiodine-avid distant metastases. The other observation is the effective use of RAI after sorafenib and radiation therapy. The patient's response to RAI probably was independent of sorafenib, as a previous study could not demonstrate the effect of sorafenib on the reinduction of RAI uptake despite control of tumor progression (21). Moreover, radioiodine uptake was not impaired by the prior radiotherapy.

In summary, we describe the first case of unresectable papillary thyroid carcinoma in which sorafenib was used as an induction therapy. Sorafenib treatment resulted in improvement of respiratory symptoms and in sufficient reduction of the tumor mass to enable total thyroidectomy and radioactive iodine treatment, providing long-term control of the disease.
